# Prognostic value of the micronucleus assay for clinical endpoints in neoadjuvant radiochemotherapy for rectal cancer

**DOI:** 10.1186/s12885-021-07914-5

**Published:** 2021-03-04

**Authors:** Leif Hendrik Dröge, Steffen Hennies, Stephan Lorenzen, Lena-Christin Conradi, Henriette Quack, Torsten Liersch, Christian Helms, Miriam Alice Frank, Markus Anton Schirmer, Margret Rave-Fränk, Tim Beißbarth, Hendrik Andreas Wolff

**Affiliations:** 1 Department of Radiotherapy and Radiation Oncology, University Medical Center Göttingen, Robert-Koch-Str. 40, 37075 Göttingen, Germany; 2 University Medical Center Göttingen, Göttingen, Germany; 3 Department of Radiology, Nuclear Medicine and Radiotherapy, Radiology Munich, 80333 Munich, Germany; 4 Institute of Medical Bioinformatics, University Medical Center Göttingen, Göttingen, Germany; 5 Department of Molecular Medicine, Bernhard Nocht Institute for Tropical Medicine, Hamburg, Germany; 6 Department of General, Visceral and Pediatric Surgery, University Medical Center Göttingen, Göttingen, Germany; 7 Department of Hematology and Medical Oncology, University Medical Center Göttingen, Göttingen, Germany; 8 Department of Radiotherapy and Radiation Oncology, University Medical Center Regensburg, Regensburg, Germany

**Keywords:** Rectal cancer, Radiochemotherapy, Cytogenetic damage, Micronucleus test, Toxicity, Survival

## Abstract

**Background:**

The question whether lymphocyte radiosensitivity is representative of patients’ response to radiotherapy (RT) remains unsolved. We analyzed lymphocyte cytogenetic damage in patients who were homogeneously treated with preoperative radiochemotherapy (RCT) for rectal cancer within clinical trials. We tested for interindividual variation and consistent radiosensitivity after in-vivo and in-vitro irradiation, analyzed the effect of patients’ and RCT characteristics on cytogenetic damage, and tested for correlations with patients’ outcome in terms of tumor response, survival and treatment-related toxicity.

**Methods:**

The cytokinesis-block micronucleus cytome (CBMNcyt) assay was performed on the peripheral blood lymphocytes (PBLCs) of 134 patients obtained before, during, at the end of RCT, and during the 2-year follow-up. A subset of PBLCs obtained before RCT was irradiated in-vitro with 3 Gy. RCT included 50.4 Gy of pelvic RT with 5-fluorouracil (5-FU) alone (*n* = 78) or 5-FU plus oxaliplatin (*n* = 56). The analyzed variables included patients’ age, gender, RT characteristics (planning target volume size [PTV size], RT technique), and chemotherapy characteristics (5-FU plasma levels, addition of oxaliplatin). Outcome was analyzed as tumor regression, patient survival, and acute and late toxicity.

**Results:**

Cytogenetic damage increased significantly with the radiation dose and varied substantially between individuals. Women were more sensitive than men; no significant age-dependent differences were observed. There was a significant correlation between the cytogenetic damage after in-vitro irradiation and in-vivo RCT. We found a significant effect of the PTV size on the yields of cytogenetic damage after RCT, while the RT technique had no effect. Neither the addition of oxaliplatin nor the 5-FU levels influenced cytogenetic damage. We found no correlation between patient outcome and the cytogenetic damage.

**Conclusions:**

We found consistent cytogenetic damage in lymphocytes after in-vivo RCT and in-vitro irradiation. Gender was confirmed as a well-known, and the PTV size was identified as a less well-known influencing variable on lymphocyte cytogenetic damage after partial-body irradiation. A consistent level of cytogenetic damage after in-vivo and in-vitro irradiation may indicate the importance of genetic factors for individual radiosensitivity. However, we found no evidence that in-vivo or in-vitro irradiation-induced cytogenetic damage is an adequate biomarker for the response to RCT in rectal cancer patients.

**Supplementary Information:**

The online version contains supplementary material available at 10.1186/s12885-021-07914-5.

## Background

The patients’ response to radiotherapy (RT) or radiochemotherapy (RCT) varies greatly. Reliable biomarkers for prediction of the individual radiosensitivity would facilitate personalized, safer exposure to irradiation and prevent underdosing of irradiation in the tumor tissue as well as high-grade normal tissue reactions [1, 2].

The question of whether lymphocyte radiosensitivity is representative of the patients’ response to RT or RCT still remains unsolved. Therapy-induced changes have been analyzed in gene expression studies (e.g., Sonis et al. [3]), apoptosis induction (e.g., Ozsahin et al. [4]), γ-H2AX techniques [5–7], and classical cytogenetics [8–10]. However, none of these approaches have consistently identified radiosensitive patients or are routinely used in the clinic. The two major cytogenetic endpoints for radiosensitivity testing purposes are chromosome aberrations and micronuclei (MN); the latter has some advantages in the practicability and multiplicity [11, 12]. Investigations in peripheral blood lymphocytes (PBLCs) after in-vitro irradiation have shown a linear increase in the MN yields as a function of the dose [13] as well as significant inter-individual differences in the response to equal irradiation doses [14]. Occasionally, the suitability of MN yields as biomarkers for the individual radiosensitivity was analyzed in clinical studies; however, the results remain controversial [10, 15–17].

We analyzed lymphocyte cytogenetic damage with the cytokinesis-block micronucleus cytome (CBMNcyt) assay [11] in secondary analyses of patients treated within prospective clinical trials at a single institution. Patients were treated with preoperative RCT for locally advanced rectal cancer (LARC), predominantly in the CAO/ARO/AIO-04-trial [18]. Herein, we tested the hypotheses of a correlation between lymphocyte cytogenetic damage after in-vivo and in-vitro irradiation and patient response to RCT, considering well-known and presumable factors, which may account for different yields in the CBMNcyt assay.

## Methods

### Patients

We analyzed 134 patients who were treated at the University Medical Center Göttingen for LARC from 11/2007 to 07/2012. Patients were recruited from three prospective trials (CAO/ARO/AIO-04 [EudraCT no.: 2006–002385-20, [18]], TransValid-KFO179/GRCSG-A [DRKS-ID: DRKS00003659] and TransValid-KFO179/GRCSG-B [EudraCT no.: 2011–004228-37]; see Table 1 for patient distribution and therapy sequence). In accordance with the respective trial protocols, treatment consisted of preoperative RCT (including pelvic RT and 5-fluorouracil (5-FU)-based chemotherapy (CT)), and highly standardized and quality-controlled TME (total mesorectal excision) surgery and histological examination in all patients. The sample size calculation was based on a previous study [12]. The Ethics Committee at the University of Göttingen approved the study, and all patients gave informed consent in written form. The investigations were conducted according to the Declaration of Helsinki principles.

**Table 1:** Patient characteristics, tumor characteristics, therapy regimen, chemotherapy and radiotherapy characteristics

**Patient characteristics**	
**Sex**	
Men	81
Women	53
**Age [y]**	
Median	67.5
Min	21
Max	87
**Weight [kg]**	
Median	80
Min	45
Max	140
**Height [m]**	
Median	1.71
Min	1.51
Max	1.93
**BMI [kg/m**^**2**^**]**	
Median	27.34
Min	17.58
Max	43.82
**Therapy regimen**	
**Clinical trials**	
CAO/ARO/AIO-04	111
TransValid-KFO179/GRCSG-A	15
TransValid-KFO179/GRCSG-B	8
**Therapy sequence**	
RCT-TME-CT	126
RCT-CT-TME	8
**CT characteristics**	
**CT regimen**	
5-FU-mono	78
FOLFOX	56
**5-FU plasma levels (AUC [mg∙h/l])**	
Median	20.37
Min	2.02
Max	100.00
**RT characteristics**	
**RT technique**	
3DCRT	87
IMRT	2
VMAT	31
3DCRT and VMAT	14
**PTV size [cm**^**3**^**]**	
Median	1414.7
Min	998.6
Max	2735.5

*RCT* radiochemotherapy, *TME* total mesorectal excision, *CT* chemotherapy, *RT* radiotherapy, *5-FU* 5-fluorouracil, *FOLFOX* 5-FU and oxaliplatin, *3DCRT* 3D-conformal radiotherapy, *IMRT* intensity modulated radiotherapy, *VMAT* volumetric modulated arc therapy, *AUC* area under the concentration-time curve, *PTV size* planning target volume size

### Radiotherapy parameters

RT was applied once daily and five times per week in 28 fractions to a reference dose of 50.4 Gy with 6 MeV or 20 MeV linear accelerator photons. Patients were positioned in the abdominal position on a belly board during the planning computed tomography and treatment. The clinical target volume (CTV) and the organs at risk were outlined on the same computed tomography images, according to the trial protocols [18], using the system Eclipse (version 8.9, Varian Medical Systems, Helsinki, Finland). The planning target volume (PTV) was defined by the addition of a 10-mm isotropic margin to the CTV. All treatment plans were calculated according to ICRU recommendations [19]. The treating radiation oncologist set the RT technique according to the individual pelvic anatomy with the aim of a high target volume conformity and low radiation exposure to the organs at risk, including 3D-conformal radiotherapy (3DCRT), intensity modulated radiotherapy (IMRT) or volumetric modulated arc therapy (VMAT).

### Patient follow-up

The tumor staging in the resected specimen was based on the sixth edition of the TNM classification [20]. The tumor regression grading (TRG) was assessed by the quantification of the ratio of tumor tissue versus fibrotic tissue (Dworak score) [21]. The grades were: grade 4 (complete tumor regression), grade 3 (fibrosis in > 50%), grade 2 (fibrosis in > 25 to 50%), grade 1 (fibrosis in ≤25%), and grade 0 (no tumor regression).

Acute organ toxicity during RCT was assessed via the National Cancer Institute Common Terminology Criteria for Adverse Events (CTCAE), version 3.0 [22]. A minimum of weekly examinations by the treating radiation oncologist were mandatory. After the completion of the therapy, patients were closely monitored for at least 2 weeks and beyond that in the case of persisting acute toxicity. Late toxicity was evaluated according to the Late effects of normal tissues (LENT-SOMA) scale [23]. Patients were monitored for late toxicity 90 days after RCT and thereafter at least annually for up to 5 years.

### CBMNcyt assay

The CBMNcyt assay [11] was performed on PBLCs obtained from patients before RCT (*n* = 134), during RCT (21.6 Gy, *n* = 128), at the end of RCT (50.4 Gy, *n* = 127) and during aftercare (1 year, *n* = 56; 2 years, *n* = 48). PBLCs obtained before RCT were additionally irradiated at 3 Gy in-vitro (*n* = 132). In-vitro irradiation, at a dose rate of 2 Gy/min, was delivered by a RS 225 X-Ray Research System (Gulmay Medical Systems, Camberley, Surrey, UK) operated at 200 kV, 15 mA and with 0.5-mm Cu filtration.

Heparinized blood samples were diluted 1:2 with 0.9% NaCl. The PBLCs were isolated by density gradient centrifugation (2400 rpm, 15 min). RPMI was added to a total volume of 50 ml. Cell division was stimulated by Phytohaemagglutinin (120 μg). After 44 h of cultivation (37 °C, 5% CO_2_), Cytochalasin B (45 μg) was added for cytokinesis-block. After an additional 28 h of cultivation (37 °C, 5% CO_2_), cytospin centrifugation (1200 rpm, 8 min) was performed, which was followed by methanol fixation, FPG staining and characterization under an optical microscope. The total levels of MN and nucleoplasmatic bridges (NPB) per 1000 binucleated lymphocytes (BNL) were assessed. The results are given as MN or NPB per single BNL, respectively [11].

### 5-FU immunoassay

The two-reagent nanoparticle agglutination assay for 5-FU was performed on blood samples taken in EDTA or heparin tubes. Because several consecutive blood samples were required at predefined times, we only collected the entire data for a subdivision of patients (*n* = 59), either receiving CT with 5-FU mono (*n* = 31; 1000 mg/m^2^/d) or 5-FU and oxaliplatin (FOLFOX, *n* = 28; 5-FU dose 250 mg/m^2^/d). Multiple specimens per patient were acquired during preoperative RCT after reaching the steady state of the plasma drug concentration (starting, at the earliest, 2 h after CT infusion set in; 5-FU mono: days 2, 4, 30, and 32; FOLFOX: days 1, 2, 8, 22, and 23). The blood plasma was isolated (centrifugation at 2200 rpm, 10 min) and stored at − 80 °C. After defreezing, the assay was performed using the “COBAS INTEGRA800”-system (Roche Diagnostics, Mannheim, Germany) and the “My5-FU-calibrator-kit” (Saladax Biomedical, Bethlehem, USA). The area under the concentration-time curve (AUC, Table 1) was calculated on the basis of the infusion duration and the measured concentration using well-established local methods [24, 25].

### Statistical analysis

The statistical tests were performed using “R” (version 3.0.2; www.r-project.org), including the packages Kendall (version 2.2) and survival (version 2.38). The differences in yields of MN and NPB during the course of RCT and during aftercare were tested by the paired one-tailed t-test for consecutive time points. The effects of gender, RT technique (3DCRT vs. any other technique) and CT (5-FU mono vs. FOLFOX) on cytogenetic damage were tested using the Mann-Whitney U-Test. The relationship of TRG and cytogenetic damage, the in-vitro vs. in-vivo yields and the effects of age, 5-FU levels, and PTV size on cytogenetic damage were tested using the rank correlation test for Kendall’s τ. *P*-values < 0.05 were considered statistically significant. Variables that showed a significant impact in univariate analysis were further studied using multivariate linear regression models. The number of MN and NPB after RCT was modeled as a function of the different influence variables to assess their significance when evaluated in combination.

The Cox regression model and Kaplan-Meier survival curves were used to estimate survival outcomes. The patient cohort was stratified into two groups according to the median of MN/BNL (after 3 Gy in-vitro irradiation: 0.242, after 50.4 Gy of RCT: 0.227) or NPB/BNL (after 3 Gy in-vitro irradiation: 0.027, after 50.4 Gy of RCT: 0.023). Recurrence-free survival was defined as the time from TME surgery to locoregional recurrence or distant metastases. Locoregional recurrence-free survival was defined as the time from TME surgery to local or regional recurrence. Distant metastasis-free survival was defined as the time from TME surgery to distant metastases. Cancer-specific survival was defined as the time from TME surgery to any death related to tumor recurrence. Non-tumor-related deaths were censored. Significance tests were performed using the Cox proportional hazards model. *P*-values, Hazard Ratios (HR) and expected 3- and 5-year survival rates, including 95%-confidence intervals, were reported.

## Results

### Cytogenetic damage after in-vivo and in-vitro irradiation

**Fig. 1:**
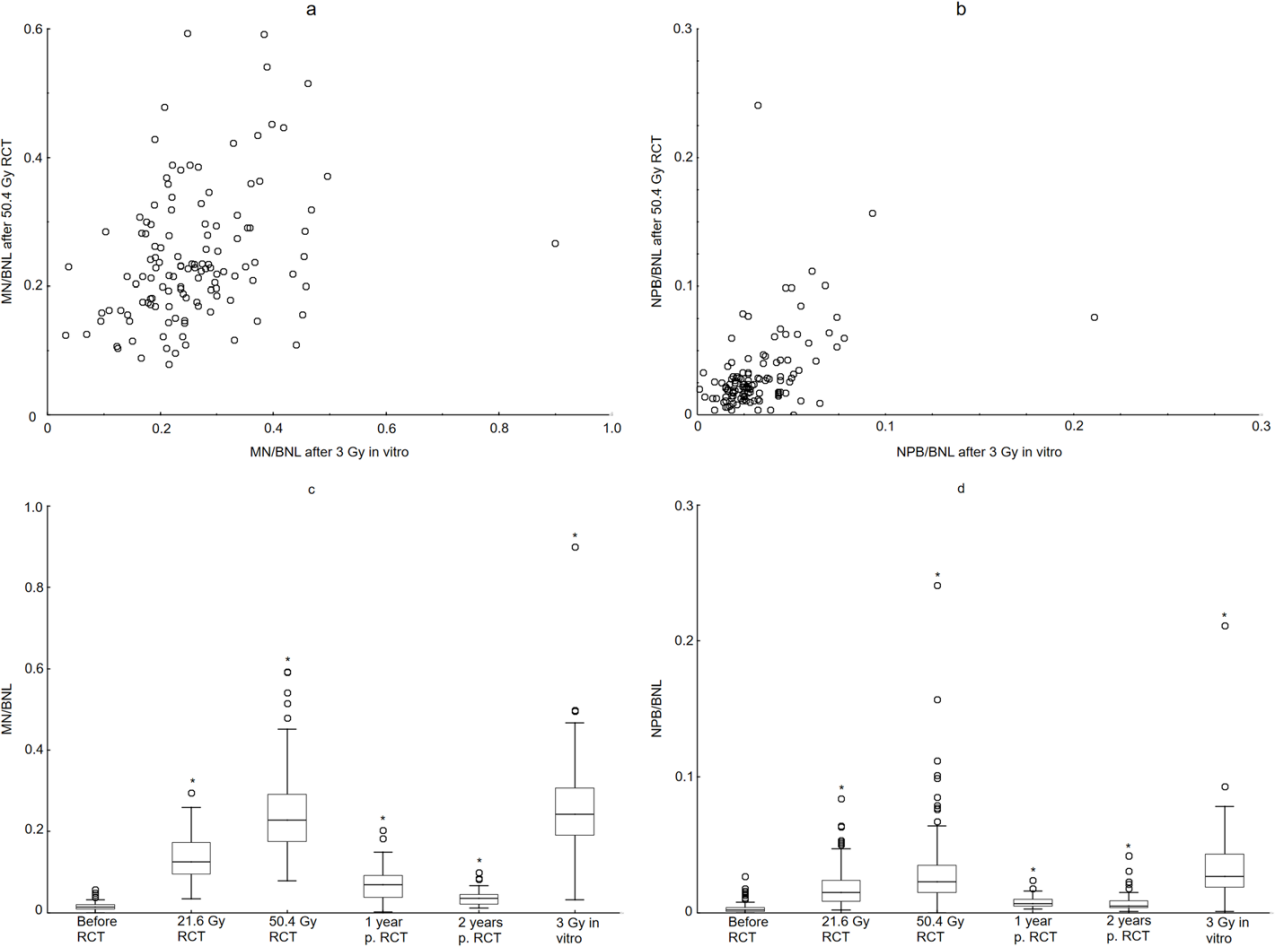
Micronuclei (MN) and nucleoplasmatic bridges (NPB) counted in binucleated lymphocytes (BNL) of rectal cancer patients. Yields after 50.4 Gy of radiochemotherapy (RCT) plotted against the yields after 3 Gy of in-vitro irradiation (**a, b**). A highly significant consistency was found between the effects of clinical RCT and the experimental in-vitro irradiation for individual patients (τ = 0.235, *p* = 0.00014) and (τ = 0.308, *p* = 7.15e-07) for MN (**a**) and NPB (**b**), respectively. The yields increased during RCT and decreased thereafter, and are still increased 2 years after RCT. Box plots for MN (**c**) and NPB (**d**). Patients had significant interindividual differences after RCT and after 3 Gy of in-vitro irradiation (**c, d**). *indicates significant differences (*p* < 0.05 with respect to baseline data)

The MN and NPB yields varied substantially between patients at all radiation doses and time points (Table 2). There was a significant correlation between the yields induced by the 3 Gy in-vitro irradiation and yields found in patients after 21.6 Gy and 50.4 Gy RCT. For the MN, we found τ = 0.168, *p* = 0.006, and τ = 0.235, *p* = 0.00011, after 21.6 Gy and 50.4 Gy RCT, respectively. For the NPB, the corresponding data were τ = 0.432, *p* < 0.00001, and τ = 0.308, p < 0.00001, after 21.6 Gy and 50.4 Gy RCT, respectively (Fig. 1a-b). In-vivo-induced cytogenetic damage (MN and NPB, please see Fig. 1c-d) increased significantly during the course of RCT (comparison of yields after 21.6 Gy and 50.4 Gy with yields before RCT, respectively). The cytogenetic damage decreased during aftercare (comparison of yields after 50.4 Gy and 1 and 2 years after RCT, respectively). Finally, the yields of MN and NPB at the time points 1 and 2 years after RCT were still significantly higher than before RCT (Fig. 1c-d).

**Table 2:** CBMNcyt assay results and the effect of gender on the MN and NPB yields

	**Women**					
	**MN/BNL**			**NPB/BNL**		
	min	max	median	min	max	median
Before RCT (0Gy)	0.005	0.057	0.018	0.000	0.018	0.002
3Gy in vitro	0.032	0.498	0.251	0.001	0.093	0.029
During RCT (21.6Gy)	0.035	0.295	0.140	0.002	0.064	0.018
End RCT (50.4Gy)	0.089	0.593	0.232	0.006	0.241	0.027
1 year after RCT	0.018	0.184	0.081	0.005	0.025	0.008
2 years after RCT	0.012	0.099	0.040	0.002	0.042	0.005
	**Men**					
	**MN/BNL**			**NPB/BNL**		
	min	max	median	min	max	median
Before RCT (0Gy)	0	0.03	0.012	0	0.027	0.002
3Gy in vitro	0.069	0.899	0.236	0.008	0.211	0.027
During RCT (21.6Gy)	0.047	0.231	0.119	0.003	0.084	0.017
End RCT (50.4Gy)	0.079	0.541	0.219	0.004	0.079	0.022
1 year after RCT	0.018	0.203	0.060	0.003	0.016	0.006
2 years after RCT	0.014	0.067	0.029	0.001	0.021	0.007

*RCT* radiochemotherapy, *MN* micronuclei, *BNL* binuclear lymphocytes, *NPB* nucleoplasmatic bridges

### No correlation between lymphocyte cytogenetic damage and patient response to RCT

Using the rank correlation test for Kendall’s τ, we found no significant correlation between TRG, acute and late organ toxicity and lymphocyte cytogenetic damage, neither for in-vitro-irradiated lymphocytes, nor for lymphocytes analysed after a full course of RCT (Table 3). Accordingly, no correlation between patient survival and lymphocyte cytogenetic damage was noticed (Table 4, Suppl. Figs. 3a-b).

**Table 3:** Tumor regression grade, acute toxicity, late toxicity (a), and analysis of the correlation between MN and NPB yields and TRG, acute and late toxicity (b). Information on tumor regression grade and late toxicity is missing in 3 and 6 patients, respectively

**a**								
	**Grade 0**	**Grade 1**		**Grade 2**		**Grade 3**		**Grade 4**
**TRG**	1	14		45		50		19
**Acute toxicity**								
Skin	50	50		22		10		0
Bladder	63	55		9		4		1
Rectum	18	65		31		16		2
Small bowel	51	54		18		5		4
**Late toxicity**								
Skin	110	16		0		0		0
Bladder	105	4		2		14		1
Rectum	88	12		19		2		5
**b**								
	**MN/BNL**				**NPB/BNL**			
	3Gy in vitro		End RCT (50.4Gy)		3 Gy in vitro		End RCT (50.4Gy)	
	Kendall’s τ [CI]	P	Kendall’s τ [CI]	P	Kendall’s τ [CI]	P	Kendall’s τ [CI]	P
**TRG**	0.09 [−0.04–0.19]	0.18	−0.02 [−0.13–0.10]	0.78	−0.06 [−0.16–0.07]	0.42	−0.12 [−0.21–0.02]	0.09
**Acute toxicity**								
Skin	0.10 [−0.01–0.18]	0.13	0.04 [−0.08–0.14]	0.56	0.00 [− 0.12–0.12]	1.00	− 0.09 [− 0.18–0.04]	0.21
Bladder	− 0.01 [− 0.11–0.10]	0.88	0.06 [− 0.06–0.16]	0.36	0.09 [− 0.04–0.18]	0.21	0.04 [− 0.08–0.13]	0.61
Rectum	0.13 [0.00–0.21]	0.06	0.02 [− 0.11–0.14]	0.82	− 0.17 [− 0.25 - -0.02]	0.01	− 0.10 [− 0.20–0.03]	0.15
Small bowel	−0.07 [− 0.17–0.05]	0.28	0.02 [− 0.10–0.13]	0.77	0.04 [− 0.07–0.14]	0.56	0.09 [− 0.04–0.18]	0.21
**Late toxicity**	0.07 [− 0.06–0.17]	0.30	−0.03 [− 0.15–0.10]	0.67	−0.07 [− 0.16–0.06]	0.33	−0.04 [− 0.14–0.08]	0.61
Skin	0.00 [− 0.08–0.08]	1.00	−0.12 [− 0.13–0.01]	0.11	0.02 [− 0.07–0.09]	0.75	0.03 [− 0.06–0.09]	0.72
Bladder	−0.03 [− 0.10–0.07]	0.73	0.01 [− 0.09–0.10]	0.89	−0.10 [− 0.014–0.03]	0.17	−0.03 [− 0–09 - 0.06]	0.68
Rectum	0.06 [− 0.06–0.14]	0.40	−0.10 [− 0.17–0.03]	0.18	0.04 [− 0.07–0.12]	0.57	0.02 [− 0.09–0.11]	0.83

*TRG* tumor regression grading, *RCT* radiochemotherapy; MN: micronuclei, *BNL* binuclear lymphocytes, *NPB* nucleoplasmatic bridges

**Table 4:** Survival data (a) and analysis of the correlation between MN and NPB yields and patient survival (b)

**a**								
	**3 years [95%-CI]**				**5 years [95%-CI]**			
**Recurrence-free survival**	74% [67–82%]				72% [64–80%]			
**Locoregional recurrence-free survival**	95% [91–99%]				92% [87–98%]			
**Distant metastasis-free survival**	74% [67–82%]				71% [63–80%]			
**Cancer-specific survival**	85% [79–92%]				76% [67–86%]			
**b**								
	**MN/BNL**				**NPB/BNL**			
	3 Gy in vitro		End RCT (50.4Gy)		3 Gy in vitro		End RCT (50.4Gy)	
	HR [CI]	P	HR [CI]	P	HR [CI]	P	HR [CI]	P
**Recurrence-free survival**	0.9 [0.4–1.7]	0.70	1.5 [0.7–3.0]	0.26	1.6 [0.8–3.3]	0.64	1.6 [0.8–3.3]	0.18
**Locoregional recurrence-free survival**	1.3 [0.3–5.6]	0.70	1.0 [0.2–3.8]	0.95	1.4 [0.4–5.8]	0.62	1.2 [0.3–4.9]]	0.78
**Distant metastasis-free survival**	0.6 [0.3–1.3]	0.21	1.3 [0.7–2.7]	0.43	1.3 [0.7–2.6]	0.46	1.9 [0.9–3.8]	0.09
**Cancer-specific survival**	0.6 [0.3–1.4]	0.22	1.3 [0.6–2.9]	0.57	1.4 [0.6–3.1]	0.47	1.9 [0.8–4.3]]	0.14

*MN* micronuclei, *BNL* binuclear lymphocytes, *NPB* nucleoplasmatic bridges, *RCT* radiochemotherapy

### Factors influencing cytogenetic damage

#### Patient-related parameters

With respect to lymphocyte damage, women were more sensitive than men. The MN yields were significantly higher for spontaneous rates before irradiation, after 21.6 Gy, after 50.4 Gy, and after the first year of aftercare (Table 2, Suppl. Fig. 1). However, we found no significant differences after 3 Gy in-vitro irradiation, and after 2 years of aftercare. The NPB were only increased after 21.6 Gy in women compared to men, but they were not increased at other time points (Table 2, Suppl. Fig. 1). The lymphocyte cytogenetic damage (MN and NPB) slightly increased with the patients’ age regardless of the time point or radiation dose tested; however, the association was not statistically significant (data not shown).

#### Radiotherapy parameters

**Fig. 2:**
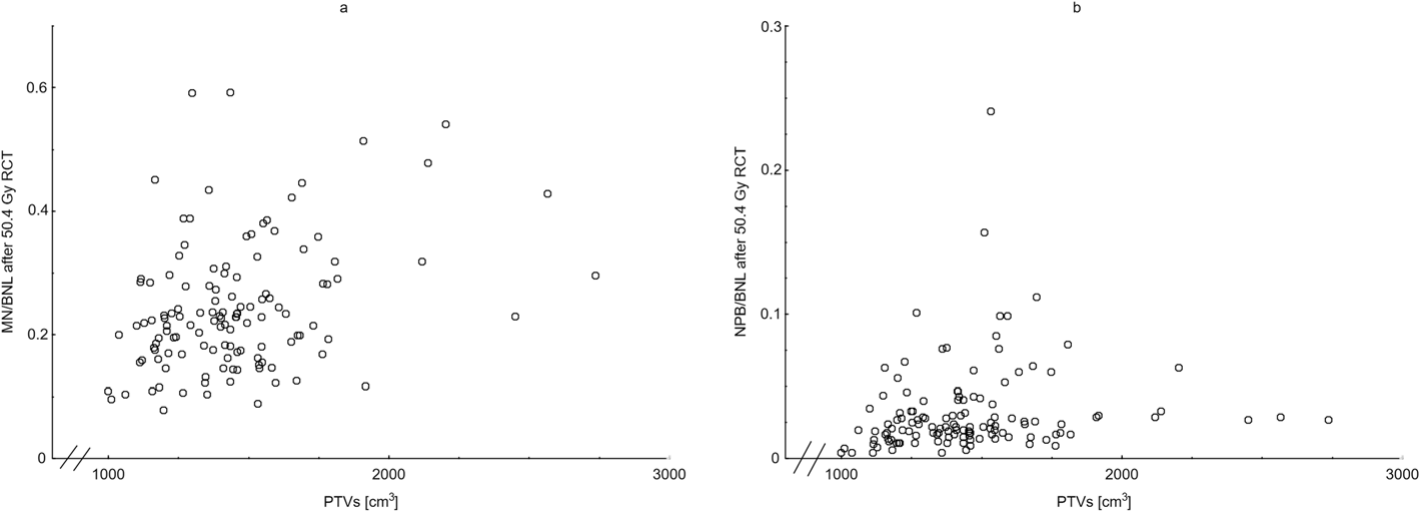
Micronuclei (MN) and nucleoplasmatic bridges (NPB) counted in binucleated lymphocytes (BNL) of rectal cancer patients. The yields after 50.4 Gy of radiochemotherapy (RCT) plotted against the individual planning target volume size. The RCT-induced cytogenetic damage as measured by MN (*p* = 0.002) (**a**) and NPB (*p* = 0.001) (**b**) increased significantly with the PTV size

We found no effect of the RT technique (3DCRT vs. IMRT/VMAT/3DCRT and VMAT) on the MN (*p* = 0.960) or NPB (*p* = 0.079) yields after 50.4 Gy of RCT, respectively. As the CTV was outlined with respect to the anatomical structures for all patients, we first considered the patient-specific influence variables on the PTV size, where height (*p* = 0.018) and weight (*p* = 0.009) showed a significant correlation with the PTV size. Gender (*p* = 0.736) and BMI (*p* = 0.212) were not correlated with the PTV size. The in-vivo-induced cytogenetic damage as measured by the MN (*p* = 0.002) and NPB (*p* = 0.001) yields after 50.4 Gy RCT increased significantly with the PTV size (Fig. 2a-b).

#### Chemotherapy

The addition of oxaliplatin to 5-FU-based RCT (*n* = 56 patients) did not increase the cytogenetic damage; the median MN yields were 0.123 vs. 0.126 after 21.6 Gy and 0.244 vs. 0.245 after 50.4 Gy (Suppl. Fig. 2). The AUC values of the 5-FU plasma drug concentration varied substantially between individual patients (Table 1), however, there was no correlation between the drug concentration and the MN (*p* = 0.528) or NPB (*p* = 0.141) yields at 50.4 Gy RCT, respectively.

### Multivariate analysis of the variables influencing the cytogenetic damage

Finally, variables with a significant impact on the MN or NPB yields after 21.6 Gy and after 50.4 Gy of RCT in the univariate analysis were further studied using multivariate models. We could exclude an influence of the height, weight and BMI of the patients on cytogenetic damage after 50.4 Gy of RCT by multivariate analysis (data not shown). There were significant correlations between the PTV size, gender, and MN yields after 3 Gy of in-vitro irradiation with the MN yields after 21.6 Gy and after 50.4 Gy RCT, respectively (Table 5). With respect to the NPB yields, the gender and yields after 3 Gy in-vitro irradiation had a significant impact on the yields after 21.6 Gy and after 50.4 Gy of RCT. However, the relationship between the PTV size and NPB yields failed to retain statistical significance in the multivariate analysis (Table 5).

**Table 5:** Univariate analysis and the subsequent multivariate linear regression models of the influential variables on the MN and NPB yields after 21.6 Gy and after 50.4 Gy of RCT

	After 21.6 Gy RCT		After 50.4 Gy RCT	
	Univariate	Multivariate	Univariate	Multivariate
	***p***-value	***p***-value	***p***-value	***p***-value
**MN yields**				
PTVs [cm^3^]	0.15	**0.04**	**0.002**	**9.4 × 10**^**−5**^
Gender	**0.015**	**0.01**	**0.017**	**0.001**
MN/BNL after 3 Gy in vitro	**0.006**	**0.01**	**1.1 × 10**^**− 4**^	**5.3 × 10**^**−5**^
**NPB yields**				
PTVs [cm^3^]	0.053	0.242	**0.001**	0.116
Gender	**0.015**	**0.007**	0.054	**0.011**
NPB/BNL after 3Gy in vitro	0.116	**6 × 10**^**−10**^	**1.0 × 10**^**−5**^	**6.2 × 10**^**− 7**^

*RCT* radiochemotherapy, *MN* micronuclei, *NPB* nucleoplasmatic bridges, *BNL* binuclear lymphocytes, *PTVs* planning target volume size

## Discussion

Herein, we report results of a CBMNcyt assay performed with secondary analyses of prospective randomized clinical trials from the German Rectal Cancer Study Group, predominantly the CAO/ARO/AIO-04 trial [18]. The data were accrued within the framework of the Clinical Research Unit 179: “Biological Basis of Individual Tumor Response in Patients with Rectal Cancer“, which aimed to enhance our understanding of the biological basis of the tumor response and to establish predictors for the response and treatment toxicity. We already reported equivalence between the CBMNcyt assay and the chromosome aberration technique in a feasibility study in which we analyzed patients (*n* = 22) treated for rectal cancer within the aforementioned framework [12]. Here, we found distinct advantages of the CBMNcyt assay [12]. Thus, we used this assay in the presented study.

In patients with LARC, a great inter-individual variation in tumor response and in RCT-related side effects has been described [26]. Suitable biomarkers could help to improve and to personalize treatment strategies [27]. The aim of the present study was to test a larger, well-defined patient cohort (*n* = 134) for a possible prognostic value of the cytogenetic damage with respect to patients’ outcome. However, we herein found no correlation between the yields of MN or NPB neither after in-vitro irradiation nor after a full course of RCT with any parameters related to patient outcome. Earlier studies reported conflicting results. Slonina et al. [16], and Finnon et al. [28] found no correlation between lymphocyte radiosensitivity, and acute and late clinically observed side effects, while Barber et al. [15] reported higher mean MN yields for patients with severe telangiectasia or fibrosis in one of three assays only. In contrast, Lee et al. [10], and Widel et al. [17] described a significant correlation between the MN frequency after in-vitro irradiation and the severity of acute and late side effects. Some authors [29–31] even found that an increase in MN frequency is predictive for the tumor reaction and patient survival.

The reason for the divergent results between the latter studies and the herein presented data are not yet clarified. All reported reliable irradiation-induced MN yields and a pronounced inter-patient heterogeneity, and some others also chose a prospective approach. Distinctive is the tumor entity, because such analyses comprising patients with LARC have not been performed before, and 5-FU and/or oxaliplatin were not given to the patients in the other studies.

The reliability of the presented data is underscored by the anticipated increase [32] in MN and NPB with age, and the greater MN frequencies in women. For age, we only found a non-significant trend at baseline and in the irradiated samples, which may be explained by the age pattern in the present study, wherein only 18% of the patients were younger than 55 years of age. Interestingly, the higher MN frequencies observed in women were not statistically significant after in-vitro irradiation. Commonly, this higher frequency is attributed to the fact that women have two copies of the X chromosome, which has a high tendency to be lost as an MN [33]. The result that only the MN frequencies and not the NPBs were increased in women supports this view. However, if the presence of two X chromosomes was the only reason, higher frequencies should have been observed after in-vivo and in-vitro irradiation. Additional host variables most likely account for gender-specific differences in the radiosensitivity, which is increasingly noted in clinical studies [34–36].

The herein described MN increase during therapy has also been found in other, mostly small, studies that analyzed miscellaneous tumor entities [37–39]. Those studies were performed on patients during RT; a concomitant CT was not included. In the present study, all patients received a 5-FU based RCT and the observed increase in cytogenetic damage was not self-evident. Chemotherapeutics and irradiation may cause interphase death or apoptosis, the magnitude of which is dose dependent. An extensive induction of interphase death or apoptosis during RCT could have caused a loss of cells with severe genomic damage, resulting in the false lowering of genomic damage [12].

The noticed persistence and partial decrease in the patients’ cytogenetic damage after the end of therapy has also been found in other cancer patients treated with external beam irradiation [39, 40], with a general decline in MN yields with increasing length of follow-up, but there is considerable variation between individuals. At 19 to 75 months of follow-up time, 7 out of 13 patients still had higher MN yields than their respective levels before therapy [40].

The irradiated volume has been described as another important variable influencing the amount of cytogenetic damage detected in lymphocytes after partial-body irradiation [41, 42]. The increase in irradiated volumes of the active bone marrow or of lymph nodes and lymph vessels might explain the correlation of irradiated target volumes and yields of cytogenetic damage in the lymphocytes [43, 44]. Again, the patient numbers were small in those studies and miscellaneous tumors were often investigated or efforts were made to achieve large volume differences [45]. Herein, we also found a significant correlation between the PTV size and MN yields after 50.4 Gy of RCT, which maintained statistical significance in the multivariate analysis and was independent from the applied RT technique (3DCRT or IMRT/VMAT/3DCRT and VMAT).

An additional important, independent influencing factor that was identified in the multivariate analysis is the level of in-vitro irradiation-induced cytogenetic damage, which correlates with the clinically observed RCT-induced damage. This correlation supports the view that variations in the radiation sensitivity are, to a certain extent, inherited, and that the individual genotype may influence the level of DNA damage in human cells. However, other factors like epigenetic features might significantly contribute to the individual radiation sensitivity and should also be studied further in this context. Eventually, our results support the perception that progress in genome-wide studies will identify risk profiles that can predict patients’ responses to radiotherapy [46–48].

Finally, based on our findings, the suitability of the CBMNcyt assay for the prediction of patient outcomes should be discussed. Firstly, the assay is relatively inexpensive [49] and our study results support the fact that the assay is suitable as a ‘biodosimeter’ for radiation exposure in individual patients [40]. Though, certain factors might limit the testing accuracy of the assay. Vral et al. discussed that the inter-individual differences in the MN background frequency might limit the accuracy of the CBMNcyt assay, whereas the specifity can be improved by the scoring of the NPB [50]. Thus, to a certain extent, our study’s results should be interpreted with caution. Secondly, the high amount of time which is necessary (only in case of manual scoring) can be considered as a disadvantage of the assay [51]. Thirdly, we did not find a correlation of the yields of cytogenetic damage with patient outcomes. This might possibly be explained by the effect of the aforementioned influencing variables (e.g., gender and PTV size) on MN and NPB yields. Thus, based on our study, we cannot provide a basis for the implementation of the CBMNcyt assay in the clinical routine to predict outcomes after RCT in case of LARC.

## Conclusions

Overall, we present a comprehensive analysis conducting the CBMNcyt assay within controlled clinical trials. Although we could implement the CBMNcyt assay in the clinical routine and could achieve reliable data, the demonstrated variation in lymphocyte radiosensitivity does not correlate with tumor or normal tissue response to radiotherapy. Therefore, we conclude that the CBMNcyt assay in case of LARC is unlikely to be predictive for patients’ outcome.

## Supplementary Information

**Additional file 1: Suppl. Figure 1.** Gender-specific comparison of micronuclei (MN) and nucleoplasmatic bridges (NPB) counted in binucleated lymphocytes (BNL). With respect to lymphocyte damage, women were more sensitive than men. The MN yields were significantly higher for spontaneous rates before irradiation, after 21.6 Gy of radiochemotherapy (RCT), after 50.4 Gy, and after the first year of aftercare. We found no significant differences after 3 Gy in-vitro irradiation, and after 2 years of aftercare. The NPB were only increased after 21.6 Gy in women compared to men, but they were not increased at other time points.

**Additional file 2: Suppl. Figure 2.** Comparison of micronuclei (MN) yields between patients who underwent radiochemotherapy (RCT) with 5-fluorouracil alone (*n* = 78) and patients who received 5-fluorouracil combined with oxaliplatin (FOLFOX, *n* = 56). The addition of oxaliplatin did not increase the cytogenetic damage. The median yields of MN were 0.123 vs. 0.126 after 21.6 Gy and 0.244 vs. 0.245 after 50.4 Gy.

**Additional file 3: Suppl. Figure 3.** a-b. There was no correlation between patient survival and lymphocyte cytogenetic damage. The Kaplan-Meier survival curves depict the cancer-specific survival (Suppl. Fig. 3a), the recurrence-free survival, the local recurrence-free survival, and the distant metastasis-free survival (Suppl. Fig. 3b). Patients were stratified according to the median of micronuclei (MN) or of nucleoplasmatic bridges (NPB), respectively, counted in binucleated lymphocytes (BNL/BL) after 50.4 Gy of radiochemotherapy (RCT). The endpoint for cancer-specific survival was any death related to tumor recurrence. Significance tests were performed using the Cox proportional hazards model.

## Data Availability

The datasets generated and/ or analyzed in the current study are available from the corresponding author by reasonable request.

## Abbreviations

RT: Radiotherapy
RCT: Radiochemotherapy
MN: Micronuclei
PBLCs: Peripheral blood lymphocytes
CBMNcyt assay: Cytokinesis-block micronucleus cytome assay
LARC: Locally advanced rectal cancer
5-FU: 5-fluorouracil
CT: Chemotherapy
TME: Total mesorectal excision
CTV: Clinical target volume
PTV: Planning target volume
3DCRT: 3D conformal radiotherapy
IMRT: Intensity modulated radiotherapy
VMAT: Volumetric modulated arc therapy
TRG: Tumor regression grading
NPB: Nucleoplasmatic bridges
BNL/BL: Binucleated lymphocytes
FOLFOX: 5-fluorouracil and oxaliplatin
