# Supplementary figures and images for: Prognostic value of the micronucleus assay for clinical endpoints in neoadjuvant radiochemotherapy for rectal cancer
Source: BMC Cancer. 2021 Mar 4;21:219. doi: 10.1186/s12885-021-07914-5 (PMC7931609; doi:10.1186/s12885-021-07914-5)

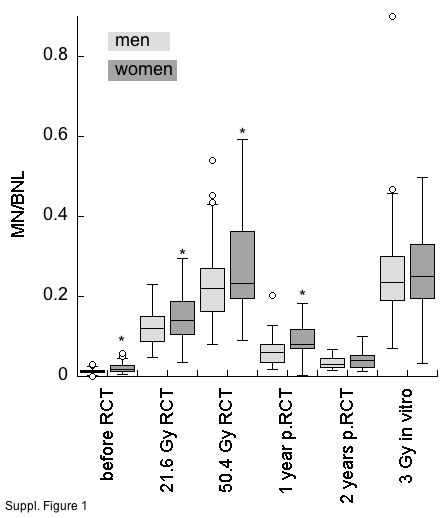

Supplement: Supplementary file 1 — Additional file 1: Suppl. Figure 1. Gender-specific comparison of micronuclei (MN) and nucleoplasmatic bridges (NPB) counted in binucleated lymphocytes (BNL). With respect to lymphocyte damage, women were more sensitive than men. The MN yields were significantly higher for spontaneous rates before irradiation, after 21.6 Gy of radiochemotherapy (RCT), after 50.4 Gy, and after the first year of aftercare. We found no significant differences after 3 Gy in-vitro irradiation, and after 2 years of aftercare. The NPB were only increased after 21.6 Gy in women compared to men, but they were not increased at other time points. [file 12885_2021_7914_MOESM1_ESM.jpg]

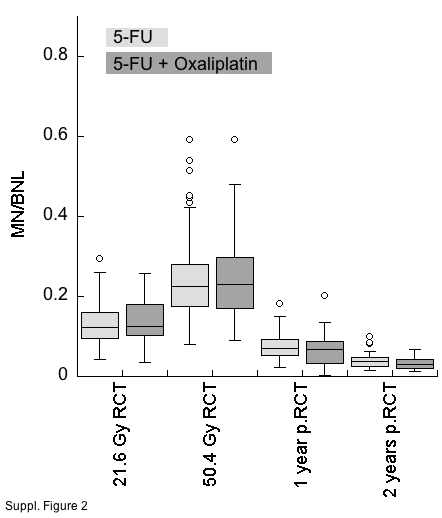

Supplement: Supplementary file 2 — Additional file 2: Suppl. Figure 2. Comparison of micronuclei (MN) yields between patients who underwent radiochemotherapy (RCT) with 5-fluorouracil alone (n = 78) and patients who received 5-fluorouracil combined with oxaliplatin (FOLFOX, n = 56). The addition of oxaliplatin did not increase the cytogenetic damage. The median yields of MN were 0.123 vs. 0.126 after 21.6 Gy and 0.244 vs. 0.245 after 50.4 Gy. [file 12885_2021_7914_MOESM2_ESM.jpg]

MN/BL after 50.4 Gy

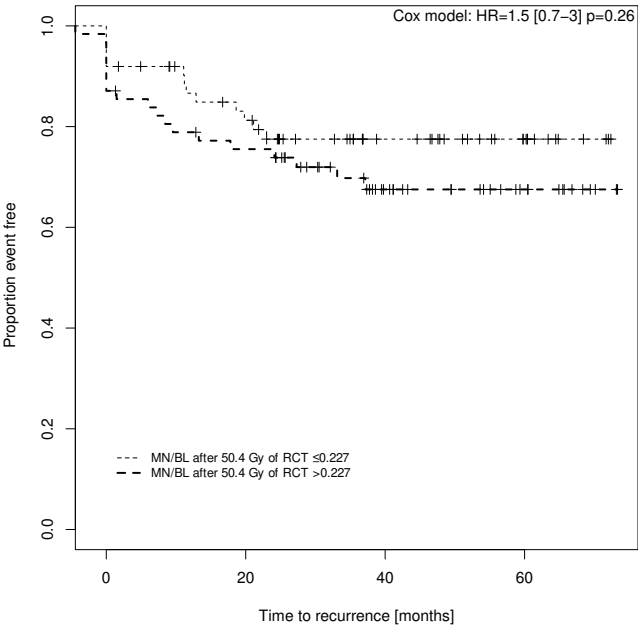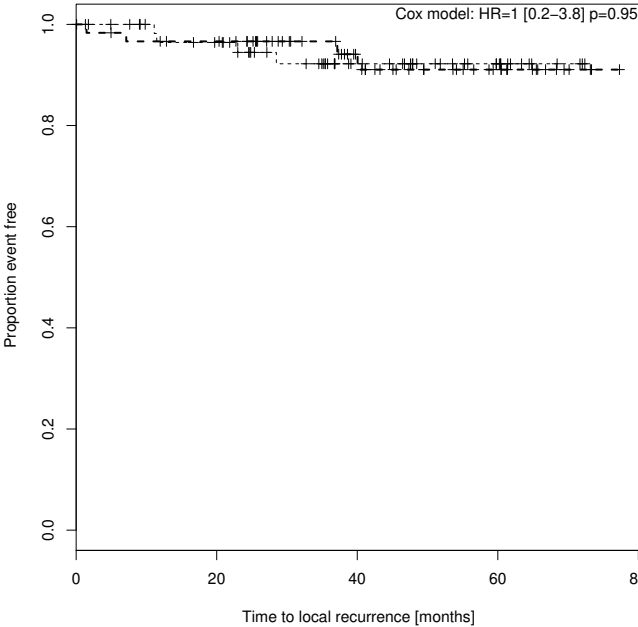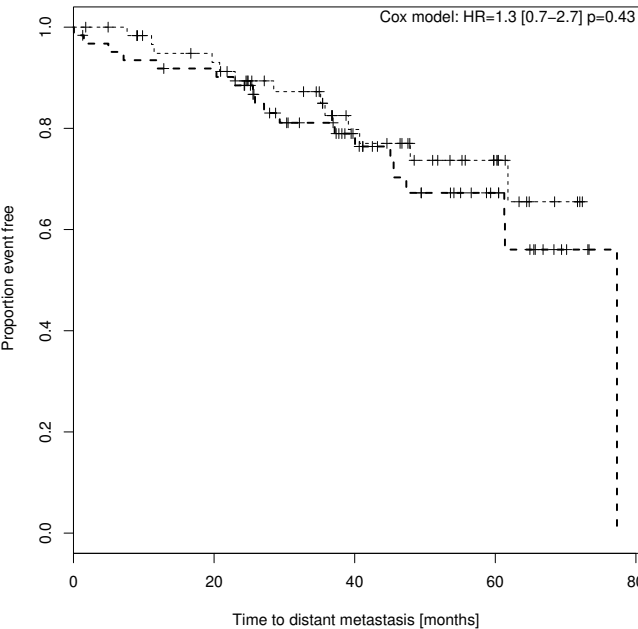

NPB/BL after 50.4 Gy

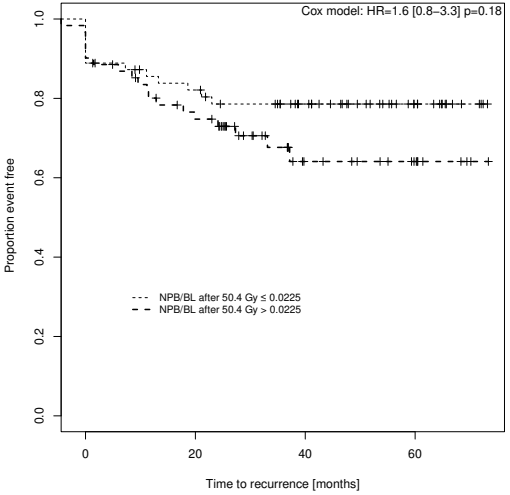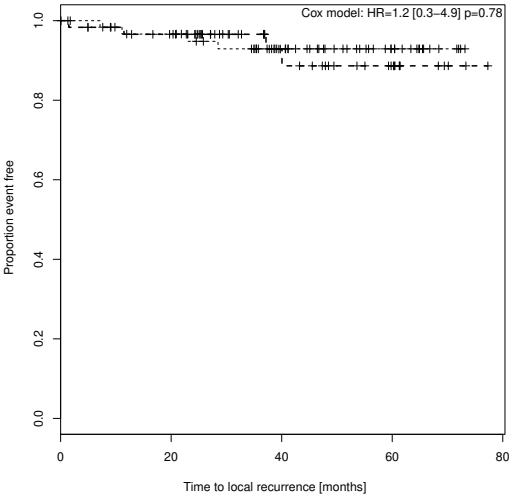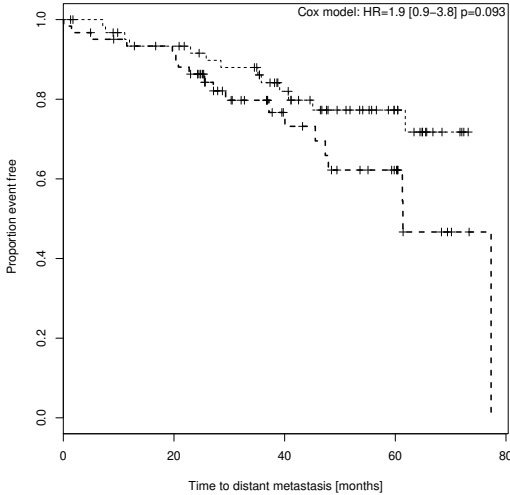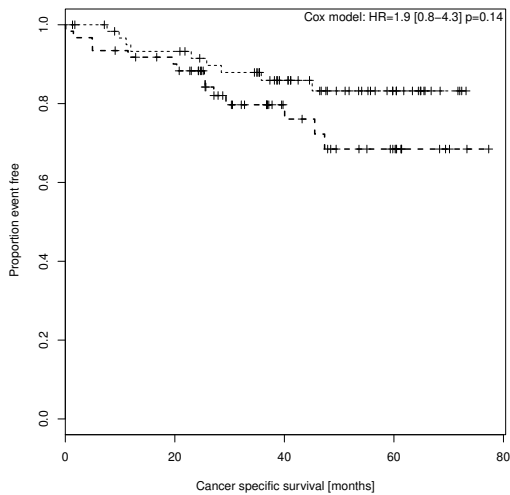

Supplement: Supplementary file 3 — Additional file 3: Suppl. Figure 3. a-b. There was no correlation between patient survival and lymphocyte cytogenetic damage. The Kaplan-Meier survival curves depict the cancer-specific survival (Suppl. Fig. 3a), the recurrence-free survival, the local recurrence-free survival, and the distant metastasis-free survival (Suppl. Fig. 3b). Patients were stratified according to the median of micronuclei (MN) or of nucleoplasmatic bridges (NPB), respectively, counted in binucleated lymphocytes (BNL/BL) after 50.4 Gy of radiochemotherapy (RCT). The endpoint for cancer-specific survival was any death related to tumor recurrence. Significance tests were performed using the Cox proportional hazards model. [file 12885_2021_7914_MOESM3_ESM.zip › Suppl. Figure 3b_revision 1R2.pdf]
