# Supplementary material for: Prognostic value of the micronucleus assay for clinical endpoints in neoadjuvant radiochemotherapy for rectal cancer
Source: BMC Cancer. 2021 Mar 4;21:219. doi: 10.1186/s12885-021-07914-5 (PMC7931609; doi:10.1186/s12885-021-07914-5)
Supplement: Supplementary file 3 — Additional file 3: Suppl. Figure 3. a-b. There was no correlation between patient survival and lymphocyte cytogenetic damage. The Kaplan-Meier survival curves depict the cancer-specific survival (Suppl. Fig. 3a), the recurrence-free survival, the local recurrence-free survival, and the distant metastasis-free survival (Suppl. Fig. 3b). Patients were stratified according to the median of micronuclei (MN) or of nucleoplasmatic bridges (NPB), respectively, counted in binucleated lymphocytes (BNL/BL) after 50.4 Gy of radiochemotherapy (RCT). The endpoint for cancer-specific survival was any death related to tumor recurrence. Significance tests were performed using the Cox proportional hazards model. [file 12885_2021_7914_MOESM3_ESM.zip › Suppl. Figure 3a_revision 1R2.pdf]

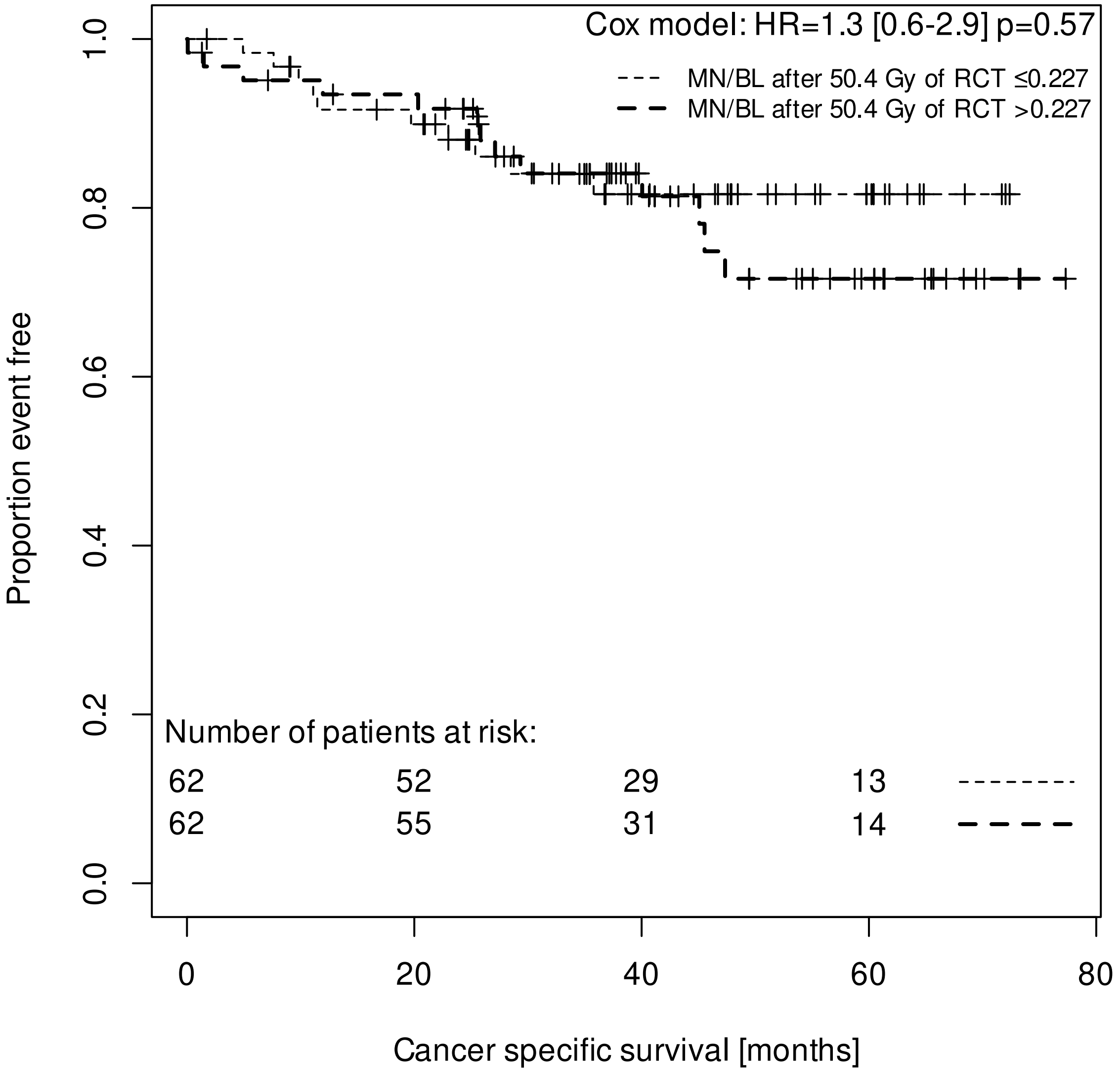

Cox model: HR=1.3 [0.6-2.9] p=0.57
MN/BL after 50.4 Gy of RCT ≤0.227
MN/BL after 50.4 Gy of RCT >0.227
Proportion event free
1.0
0.8
0.6
0.4
0.2
0.0
Number of patients at risk:
62 52 29 13
62 55 31 14
0
20
40
60
80
Cancer specific survival [months]
